# Association Between Respiratory Alkalosis and the Prognosis of COVID-19 Patients

**DOI:** 10.3389/fmed.2021.564635

**Published:** 2021-04-26

**Authors:** Chenfang Wu, Guyi Wang, Quan Zhang, Bo Yu, Jianlei Lv, Siye Zhang, Guobao Wu, Shangjie Wu, Yanjun Zhong

**Affiliations:** ^1^Critical Care Medicine, The Second Xiangya Hospital, Central South University, Changsha, China; ^2^Critical Care Medicine, The First Hospital of Changsha, Changsha, China; ^3^Department of Respiratory, The Second Xiangya Hospital, Central South University, Changsha, China

**Keywords:** COVID-19, respiratory alkalosis, acid-base disorder, severity, biomarker

## Abstract

**Aim:** The aim of the study was to describe the clinical characteristics of patients with or without respiratory alkalosis, and analyze the relationship of respiratory alkalosis and the outcome of adult coronavirus disease 2019 (COVID-19) patients.

**Methods:** Clinical and laboratory data of adult COVID-19 patients in a single center in China, were retrospectively collected and analyzed. The Kaplan-Meier (KM) curve and cox regression were adopted to analyze the association between respiratory alkalosis and prognosis of COVID-19 patients.

**Results:** Of 230 adult COVID-19 patients, 66 patients (28.7%) had respiratory alkalosis on admission. Of 66 patients, the median age was 53 years old (range, 21–84 years), and 43 (65.2%) were female. Compared with those without respiratory alkalosis, patients with respiratory alkalosis were significantly older (*P* = 0.002), had a higher proportion of female (*P* = 0.004), and showed higher ratios of underlying diseases including hypertension (*P* = 0.023) and cardiovascular disease (*P* = 0.028). Moreover, they demonstrated higher proportion of severe events (*P* = 0.001). Patients with respiratory alkalosis had a higher possibility of developing severe events compared with those without respiratory alkalosis (Log Rank *P* = 0.001). After adjusting for gender, age, and comorbidities, patients with respiratory alkalosis still showed significantly elevated risks of developing to severe cases (HR 2.445, 95% CI 1.307–4.571, *P* = 0.005) using cox regression analyses.

**Conclusions:** Respiratory alkalosis as a common acid—base disorder in COVID-19 patients, was associated with a higher risk of developing severe event.

## Introduction

Coronavirus Disease 2019 (COVID-19) caused by Severe Acute Respiratory Syndrome Coronavirus 2 (SARS-CoV-2), was first reported in Wuhan, China, at December 2019 (1–5), has spread widely and rapidly around the world (6, 7). The World Health Organization (WHO) has declared COVID-19 as a global pandemic (8). How to decrease mortality and improve the prognosis of COVID-19 has become a global problem.

Respiratory alkalosis as the most common acid—base imbalance in clinical practice, is generally induced by a process involving hyperventilation, which include hypoxemic causes, pulmonary diseases and central diseases. Pulmonary diseases include pneumonia, acute asthma and chronic obstructive pulmonary disease (COPD) exacerbations (9, 10). Furthermore, respiratory alkalosis is considered to be associated with adverse outcomes (11). Some COVID-19 patients showed hypoxemia and dyspnea, but the incidence of respiratory acid-base disorders and their relations with the prognosis of COVID-19 remained unknown. To address this question, we sought to present the clinical characteristics of COVID-19 patients with or without respiratory alkalosis, and analyze the association between respiratory alkalosis and the outcomes of COVID-19 patients.

## Materials and Methods

### Study Design and Participants

This case series was approved by The Institutional Ethics Board of The Second Xiangya Hospital of Central South University (No. 2020001). Adult laboratory-confirmed COVID-19 patients admitted to Public Health Treatment Center of Changsha, China from January 17 to March 14, 2020, were enrolled.

### Primary and Second Endpoints

The primary endpoint was the severity of COVID-19, and the second endpoints were the mortality, virus shedding time, and length of hospital stay. Severe cases of COVID-19 were defined as meeting any of the following criteria: (1) respiratory rate ≥ 30 /min; (2) oxygen saturation < 93%; (3) PaO_2_/FiO_2_ ≤ 300 mmHg; (4) lung lesions progressed >50% within 24–48 h; (5) mechanical ventilation was implemented; (6) shock; (7) intensive care was required because of other organ dysfunction (12). At least two consecutive nucleic acid tests were performed after remission. Virus shedding duration was defined as time between symptom onset (or the diagnosed date for asymptomatic patients) and the first negative sample of SARS-CoV-2 without any positive sample thereafter.

### Data Collection

The medical records of patients were carefully collected by two authors, separately. Demographic, symptoms, underlying disease, laboratory, and outcome data were collected. All patients underwent blood gas analysis within the first day after admission and the first blood gas analysis results after admission were collected. In case of PH > 7.45, PaCO_2_ < 35 mmHg in arterial blood gas was considered as respiratory alkalosis, and in case of pH < 7.35, PaCO_2_ > 45 mmHg was defined as respiratory acidosis (13).

### Statistical Analysis

We used median with range and Mann-Whitney-test to depict and analyze the data. The χ^2^-test or Fisher's exact-test was utilized to compare the differences of the categorical variables. Baseline variables with significant differences between respiratory alkalosis and no respiratory alkalosis groups were included in the univariate and multivariate analyses. The Kaplan-Meier (KM) curve and the Log Rank-test were applied to estimate the cumulative proportion of severe events in non-severe patients after admission according to the respiratory alkalosis groups. Multivariate analysis was performed using the cox regression model to determine the association between respiratory alkalosis and the severe event with the hazards ratio (HR) and 95% confidence interval (95% CI) being reported. All analyses were performed using IBM SPSS version 26 software.

## Results

Two hundred and thirty adult patients with laboratory-confirmed SARS-CoV-2 infection by March 14, 2020, in Changsha, China, were included in our study, of whom none had respiratory acidosis and 66 patients (28.7%) had respiratory alkalosis on admission.

Of the 66 patients with respiratory alkalosis, the median age was 53 years old (range, 21–84 years), and 43 (65.2%) were women. Hypertension, diabetes and cardiovascular disease were the most common comorbidities. The most common symptoms were fever, cough, anorexia and fatigue. Most (97.0%) patients showed pulmonary exudative lesion in chest computed tomographic (CT) scan, of which 32 (48.5%) patients showed ground glass opacity in the lungs (Table 1).

**Table 1:** Baseline characteristics of COVID-19 patients with or without respiratory alkalosis.

	**Respiratory alkalosis (*n* = 66)**	**No respiratory alkalosis (*n* = 164)**	***P*-value**
Sex (male/female)	23/43	92/72	**0.004**
Age, median (range), y	53 (21–84)	42 (19–82)	**0.002**
**Comorbidity**			
Hypertension (*n*, %)	16 (24.2)	20 (12.2)	**0.023**
Cardiovascular disease (*n*, %)	6 (9.1)	3 (1.8)	**0.028**
Diabetes (*n*, %)	6 (9.1)	9 (5.5)	0.480
Cerebrovascular disease (*n*, %)	3 (4.5)	3 (1.8)	0.358
**Symptoms**			
Fever (*n*, %)	58 (87.9)	117 (71.3)	**0.008**
Fatigue (*n*, %)	41 (62.1)	66 (40.2)	**0.003**
Cough (*n*, %)	55 (83.3)	134 (81.7)	0.771
Anorexia (*n*, %)	40 (60.6)	75 (45.7)	0.041
Chills (*n*, %)	9 (13.6)	20 (12.2)	0.766
Myalgia (*n*, %)	10 (15.2)	14 (8.5)	0.138
Dyspnea (*n*, %)	33 (50.0)	49 (29.9)	**0.004**
Expectoration (*n*, %)	34 (51.5)	72 (43.9)	0.295
Pharyngalgia (*n*, %)	9 (13.6)	21 (12.8)	0.756
Diarrhea (*n*, %)	16 (24.2)	37 (22.6)	0.784
Nausea (*n*, %)	12 (18.2)	18 (11.0)	0.142
Dizziness (*n*, %)	13 (19.7)	16 (9.8)	**0.040**
Headache (*n*, %)	12 (18.2)	19 (11.6)	0.185
Vomiting (*n*, %)	14 (21.2)	12 (7.3)	**0.003**
Abdominal pain (*n*, %)	4 (6.1)	4 (2.4)	0.338
Chest CT positive rate (*n*, %)	64 (97.0)	156 (95.1)	0.792
Chest CT with ground-glass change (*n*, %)	32 (48.5)	77 (47.0)	0.833
Severe cases (*n*, %)	22 (33.3)	23 (14.0)	**0.001**
Length of hospital stay, median (range), days	16 (5–40)	16 (5–41)	0.553
Virus shedding duration, median (range), days	17 (6–43)	19 (3–59)	0.458
Mortality (*n*, %)	2 (3.0)	0 (0)	0.081

*P < 0.05 was considered statistically significant (marked in bold)*.

*COVID-19, coronavirus disease 2019*.

Laboratory findings for patients with respiratory alkalosis were shown in Table 2. On admission, the medium number of white blood cell count, lymphocyte percentage and lymphocyte count were in the normal range. C-reactive protein and erythrocyte sedimentation rate significantly elevated. Median levels of indicators of liver, kidney and myocardial injury were not obviously abnormal.

**Table 2:** Laboratory findings of COVID-19 patients with or without respiratory alkalosis.

	**Respiratory alkalosis (*n* = 66)**	**No respiratory alkalosis (*n* = 164)**	***P-*value**
White blood cell count, × 10^9^/L, median (range)	4.2 (0.8–10.4)	4.6 (1.5–13.4)	0.164
Lymphocyte count, × 10^9^/L, median (range)	0.9 (0.1–3.7)	1.2 (0.2–3.2)	**0.001**
Lymphocyte %, median (range)	24.0 (5.5–46.6)	27.5 (2.1–61.1)	**0.015**
Alanine aminotransferase, U/L, median (range)	22.4 (8.1–93.7)	18.8 (2.6–87.7)	**0.007**
Aspartate aminotransferase, U/L, median (range)	27.7 (15.1–82.1)	23.2 (2.0–78.8)	**0.000**
Total bilirubin, μmol/L, median (range)	10.4 (5.1–162.1)	11.0 (4.0–38.2)	0.873
C-reactive protein, mg/L, median (range)	24.0 (0.2–101.9)	12.0 (0.1–101.9)	**0.000**
Erythrocyte sedimentation rate, mm/h, median (range)	53.0 (3.0–143.0)	37.0 (1.0–114.0)	**0.012**
Procalcitonin, ≥0.05 ng/ml, No. (%)	24 (36.4)	39 (23.8)	0.053
Creatinine, μmol/L, median (range)	46.8 (20.6–110.3)	53.4 (21.9–255.7)	0.058
Creatine kinase, U/L, median (range)	68.4 (11.3–986.4)	76.1 (17.4–599.6)	0.882
Creatine kinase-MB, U/L, median (range)	10.1 (1.0–221.7)	9.5 (0.3–82.8)	0.758

*P < 0.05 was considered statistically significant (marked in bold)*.

*COVID-19, coronavirus disease 2019*.

Compared with patients without respiratory alkalosis, patients with respiratory alkalosis were significantly older and had a higher proportion of female. They also showed higher ratios of underlying diseases including hypertension and cardiovascular diseases. Additionally, they had lower lymphocyte proportion, decreased lymphocyte counts, as well as higher levels of C-reactive protein and erythrocyte sedimentation rate (Table 2). Most importantly, patients with respiratory alkalosis showed significantly higher proportion of severe events (Table 1).

**Figure 1:**
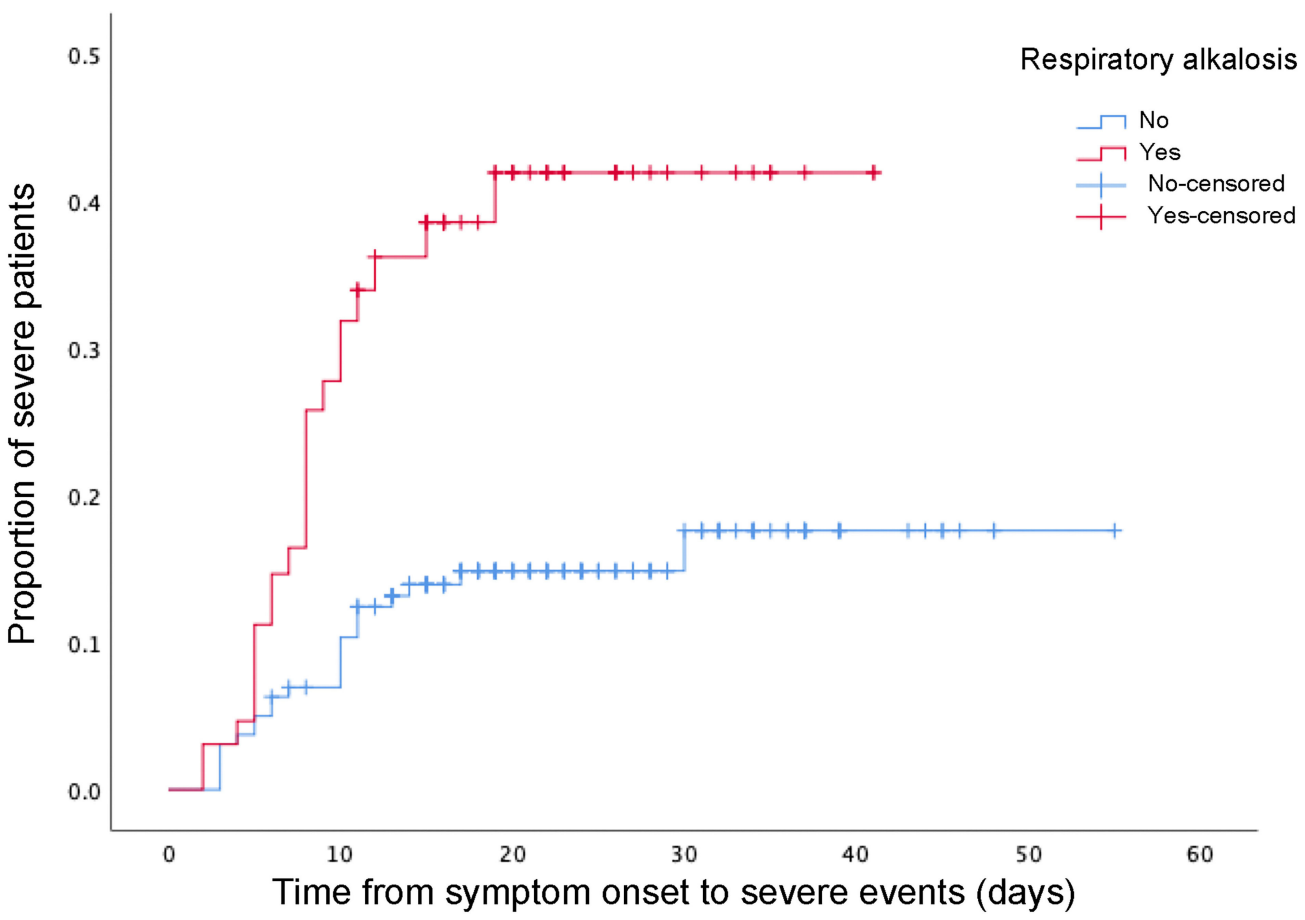
The time-dependent risk of reaching to severe events in COVID-19 patients with or without respiratory alkalosis using Kaplan-Meier analysis and log rank-test. COVID-19, coronavirus disease 2019.

Furthermore, patients with respiratory alkalosis had a higher possibility of developing severe events compared with those without respiratory alkalosis (Log Rank *P* = 0.001, Figure 1). After adjusting for gender, age and common comorbidities (cardiovascular disease and hypertension), patients with respiratory alkalosis still showed significantly elevated risks of developing to severe cases than those without respiratory alkalosis (HR 2.445, 95% CI 1.307–4.571, *P* = 0.005) (Table 3).

**Table 3:** Multivariate Cox regression analysis for severe events of adult COVID-19 patients.

**Variables**	**HR**	**95% confidence interval**	***P-*value**
Respiratory alkalosis	2.445	1.307–4.571	**0.005**
Age (≥60 y)	1.021	0.999–1.044	0.062
Gender (male)	1.973	1.056–3.588	**0.033**
Hypertension	1.927	0.946–3.924	0.071
Cardiovascular disease	1.355	0.493–3.719	0.556

*P < 0.05 was considered statistically significant (marked in bold)*.

*COVID-19, coronavirus disease 2019; HR, Hazard Ratio*.

However, in terms of virus shedding duration and length of hospital stay, patients with respiratory alkalosis showed no obvious differences (Table 1). Because hyperventilation reactions due to hypoxia may be different from gender, age and comorbidities (14), we performed a subgroup analysis on these factors for virus shedding duration and length of hospital stay. Respiratory alkalosis seemed to have no effect on virus shedding duration and length of hospital stay in both men and women, as well as subgroups of common comorbidities. Moreover, subgroup analysis on age showed in non-elderly (*P* = 0.045) but not elderly patients demonstrated shorter virus shedding duration in patients with respiratory alkalosis than those without (Table 4).

**Table 4:** Subgroup analysis of influence of respiratory alkalosis on the virus shedding duration and length of hospital stay.

	**Virus shedding duration (days)**		***P-*value**	**Length of hospital stay (days)**		***P-*value**
	**Respiratory alkalosis (*n* = 66)**	**No respiratory alkalosis (*n* = 164)**		**Respiratory alkalosis (*n* = 66)**	**No respiratory alkalosis (*n* = 164)**	
**Gender**						
Male	15 (10–39)	18 (6–59)	0.369	13 (5–36)	16 (5–41)	0.221
Female	17 (6–43)	19 (3–53)	0.824	17 (5–40)	15.5 (5–40)	0.830
**Age**						
Elderly^*^	22 (9–43)	19.5 (4–43)	0.326	19 (6–40)	17 (6–37)	0.349
Non-elderly^#^	15 (6–39)	18 (3–59)	**0.045**	13 (5–35)	15 (5–41)	0.091
**Hypertension**						
No	16 (6–43)	19 (3–59)	0.473	15.5 (5–40)	15 (5–41)	0.665
Yes	18 (12–38)	18 (6–45)	0.648	16.5 (5–37)	19.5 (7–41)	0.336
**Diabetes**						
No	16.5 (6–43)	19 (3–59)	0.521	15.5 (5–40)	16 (5–41)	0.477
Yes	16.5 (9–34)	23 (6–43)	0.776	18 (6–32)	17 (7–30)	1.000
**Cardiovascular diseases**						
No	16 (6–43)	18 (3–59)	0.210	16 (5–40)	16 (5–41)	0.458
Yes	31 (21–39)	22 (19–29)	0.250	22 (12–35)	23 (19–27)	0.786

*P < 0.05 was considered statistically significant (marked in bold)*.

* *≥60 years*,

# *<60 years*.

*COVID-19, coronavirus disease 2019*.

## Discussion

To our knowledge, this is the first article to explore the respiratory acid-base situation of COVID-19 patients and evaluate the effect of respiratory alkalosis on the prognosis. About 28.7% patients presented respiratory alkalosis on admission, who were more likely to be severe and die. Meanwhile, respiratory alkalosis was significantly associated with the risk for severe events of COVID-19 patients.

Most (65.2%) COVID-19 patients with respiratory alkalosis were female, which is consistent with other studies. Previous studies showed PCO_2_ was lower in women than men (15). Compared to men, women were more prone to have a compensated respiratory alkalosis, which may be the role of hormones such as progesterone (14, 16). However, respiratory alkalosis was associated with severe events of COVID-19 patients both in male and female in this study.

In this study, COVID-19 patients with respiratory alkalosis had a higher risk of severe events, but the reason is not very clear. Generally, hypoxic stimulation leads to hyperventilation in an attempt to correct hypoxia at the expense of a CO_2_ loss in pulmonary diseases (17). Although some patients did not show significant hypoxemia at the early stage, if respiratory alkalosis occurred, they may already have a compensatory hyperventilation and deteriorate soon. Therefore, we believe that COVID-19 patients with respiratory alkalosis need to be given adequate monitoring even though they did not show hypoxemia yet.

According to current reports (4, 18), deaths of COVID-19 patients mainly occur in severe cases, which indicates that the mortality of COVID-19 patients is closely related to the disease severity. Since there were only 2 deaths in this study, we did not perform association analysis on respiratory alkalosis and mortality, but the risk of developing severe event and respiratory alkalosis were significantly related. It suggests that respiratory alkalosis may be also a risk factor for death, but larger sample size studies are needed to verify this conclusion.

Our study has several limitations. First, some patients with respiratory alkalosis coexisted with other acid-base imbalances, which may lead some bias to the outcomes. In the future research, other acid-base imbalances also need to be studied in greater detail. Second, this study only focused on the acid-base status at the time of admission, and further analysis of the dynamic acid-base status may be needed in the future. Third, in general, most cases of respiratory alkalosis are secondary to hypoxia. However, this study did not include oxygenation index data, due to lack of some data, which may affect the analysis of the causes of respiratory alkalosis. Forth, this was a retrospectively single center study with a small sample size. Therefore, well-designed studies with large sample size were needed to demonstrate the conclusion.

## Conclusions

To the best of our knowledge, this is the first report about respiratory acid-base imbalance in COVID-19 patients. As a common acid-base disorder in COVID-19 patients, respiratory alkalosis was associated with a higher possibility of developing severe events.

## Data Availability Statement

The datasets presented in this article are not readily available because they need permission from local health and disease control authorities. Requests to access the datasets should be directed to zhongyanjun@csu.edu.cn.

## Ethics Statement

The studies involving human participants were reviewed and approved by The Institutional Ethics Board of The Second Xiangya Hospital of Central South University (No. 2020001). Written informed consent for participation was not required for this study in accordance with the national legislation and the institutional requirements.

## Author Contributions

GWa and CW developed the study design and conducted the analyses. YZ interpreted the results, reviewed the study design, and performed the statistical analyses. QZ and BY acquired patient demographic and clinical data. JL, GWu, SW, and SZ participated in interpreting the clinical results and reviewed the manuscript. All authors have read and approved the final version.

## Conflict of Interest

The authors declare that the research was conducted in the absence of any commercial or financial relationships that could be construed as a potential conflict of interest.
